# Egg intervention effect on linear growth no longer present after two years

**DOI:** 10.1111/mcn.12925

**Published:** 2019-12-17

**Authors:** Lora L. Iannotti, Melissa Chapnick, Jennifer Nicholas, Carlos Andres Gallegos‐Riofrio, Patricia Moreno, Katherine Douglas, David Habif, Yuhan Cui, Christine Stewart, Chessa K. Lutter, William F. Waters

**Affiliations:** ^1^ Institute for Public Health, Brown School Washington University in St. Louis St. Louis Missouri; ^2^ School of Medicine, Pediatric Radiology, Mallinckrodt Institute of Radiology Washington University in St. Louis St. Louis Missouri; ^3^ Instituto de Investigación en Salud y Nutrición, Diego de Robles y Via Interoceánica Universidad San Francisco de Quito Quito Ecuador; ^4^ Department of Nutrition University of California Davis Davis California; ^5^ PAHO, Food Security and Agriculture RTI International Washington DC United States; ^6^ School of Public Health University of Maryland College Park Maryland

**Keywords:** animal source foods, cohort study, complementary feeding, complementary foods, Ecuador, egg nutrition, infant growth, preschool children, stunting

## Abstract

The Lulun Project, a randomized controlled trial conducted in 2015, found that one egg per day for 6 months during early complementary feeding reduced stunting by 47% and increased linear growth by 0.63 length‐for‐age Z (LAZ). This follow‐up cohort study (Lulun Project II) aimed to test whether the growth effect remained in the egg intervention group compared with the control group after approximately 2 years. Mothers or caregivers from the Lulun Project were recontacted and recruited for this study. Enumerators collected data on socio‐economic and demographic factors, 24‐hr frequency of dietary intakes, morbidities, and anthropometric measures of height, weight, and head circumference using World Health Organization protocols. Statistical analyses followed the same analytical plan as Lulun Project, applying generalized linear models and regression modelling to test group differences in height‐for‐age *z* (HAZ) from LAZ at Lulun Project endline, and structural equation modelling for mediation. One hundred thirty‐five mother–child dyads were included in Lulun II, with 11% losses to follow‐up from endline Lulun Project. Growth faltering across all children was evident with HAZ −2.07 ± 0.91 and a stunting prevelance of 50%. Regression modelling showed no difference between egg and control groups for the HAZ outcome and other anthropometric outcomes, and significant declines in HAZ from endline Lulun Project in the egg intervention are compared with control groups. Current dietary egg intake, however, was associated with reduced growth faltering in HAZ from Lulun Project endline to Lulun Project II, independent of group assignment and through mediation, explaining 8.8% of the total effect. Findings suggest the need for a longer intervention period and ongoing nutrition support to young children during early childhood.

**List of Abbreviations**
ASF: animal source foods
BMIz: body mass index *z* score
DHA: docosahexaenoic acid
DMA: dimethyl acetal
GLM: generalized linear modelling
HAZ: height‐for‐age *z* score
HCZ: head circumference *z* score
LAZ: length‐for‐age *z* score
LNS: lipid‐based nutrient supplement
NNIPS: Nepal Nutritional Intervention Project‐Sarlahi
RCT: randomized controlled trial
TMAO: trimethyl amine *N*‐oxide
WASH: water, sanitation, and hygiene
WAZ: weight‐for‐age *z* score
WLZ: weight‐for‐length *z* score
WHZ: weight‐for‐height *z* score

**Key messages**

- Lulun Project II was a follow‐up cohort study to the Lulun Project, an randomized controlled trial carried out in 2015 in Ecuador that found one egg per day for 6 months during early complementary feeding reduced stunting by 47%.
- Group differences in height‐for‐age *z* score were no longer present 2 years after the completion of the Lulun Project egg intervention period; growth faltering was highly prevalent among all children in the follow‐up study showing a stunting prevalence of 50%.
- In regression modelling, contemporary egg consumption was associated with reduced growth faltering from endline Lulun Project to Lulun II, independent of group assignment and through mediation, explaining 8.8% of the total effect.
- Eggs can provide a critical bridge to bolster growth in highly vulnerable periods, but ongoing attention to overall diet quality and supportive environmental conditions should be sustained as a child ages.

## 1 INTRODUCTION

Animal source foods (ASF) provide crucial nutrients in highly bioavailable forms to young children during the complementary feeding period (Iannotti, 2018). These foods, long part of our evolutionary past, likely played a central role for increasing body and brain size at particular junctures in hominin history (Kuipers, Joordens, & Muskiet, 2012). However, ASF can be expensive relative to other foods and inaccessible to low‐resource households (Headey & Alderman, 2019). Eggs may be relatively more affordable ASF depending on context and highly nutritious complementary food. Eggs offer a holistic package of nutrients intended to promote rapid infant growth and development (Iannotti, Lutter, Bunn, & Stewart, 2014). The Lulun Project leveraged the opportunity to test the effects of introducing eggs early in the complementary feeding period on infant and young child growth.

From March to December 2015, we conducted a randomized controlled trial (RCT) in the highlands of Ecuador to test the efficacy of one egg per day for six months introduced early in complementary feeding period from 6 to 9 months (Lulun Project). The context and population were selected in part only to due to the high prevalence of stunting but also because formative research revealed high acceptability and cultural value placed in eggs (Gallegos‐Riofrío et al., 2018). Stunting prevalence was reduced by 47% (prevalence ratio, 0.53; 95% CI [0.37, 0.77]), and length‐for‐age *z* score (LAZ) increased by 0.63 (95% CI [0.38, 0.88]; Iannotti et al., 2017). Reinforcing the growth findings was the significant increases in biomarker concentrations of choline and docosahexaenoic acid among others, with similarly large effect sizes (Iannotti et al., 2017). Other nutrients and metabolites, such as Vitamin B12 and retinol, were not significantly increased, however (Iannotti, Lutter, Waters, et al., 2017).

Few studies of early childhood interventions have followed children beyond the intervention period. Findings from two trials in Guatemala and Mexico suggest long‐term effects from early nutrition interventions on growth and other development parameters (Fernald, Gertler, & Neufeld, 2009; Rivera, Martorell, Ruel, Habicht, & Haas, 1995). A more recent study in Bangladesh followed children ages 40–52 months from a lipid‐based nutrient supplementation trial during pregnancy, lactation and early childhood (6–24 months), found no long‐term sustained effects on anthropometric outcomes except in subgroup analyses (Dewey, Mridha, Matias, Arnold, & Young, 2017). In view of the large effect size found in the Lulun Project, we hypothesized that some portion of this effect would remain and that height‐for‐age *z* (HAZ) would be greater in the egg group compared with the control.

## 2 METHODS

### 2.1 Study design and participants

Lulun Project II was a longitudinal follow‐up study of the Lulun Project, which was centred around an RCT conducted between March and December 2015 in Cotopaxi Province, Ecuador. Located approximately 90 km south of Quito, Cotopaxi is largely rural, with low population density. Approximately 72% of the population identify as *mestizo* (mixed race), and 22% as indigenous (Instituto Ecuatoriano de Encuestas y Censo (INEC), 2011). The word “lulun” means egg in Kichwa (or Quechua) and was intentionally selected as a result of the social marketing strategy that accompanied the RCT, which facilitated acceptance, and enhanced participation and ownership in the project communities (Gallegos‐Riofrío et al., 2018).

Details of the original RCT are described elsewhere (Iannotti, Lutter, Stewart, et al., 2017). In brief, mother (or other caregiver)–child dyads were recruited during the early complementary feeding period. Infants were eligible if aged 6–9 months, singleton birth, and healthy and excluded if congenital heart condition, severe malnutrition, or egg allergies were present. Infants were randomized into control (standard of care) and egg intervention groups (1 egg per day for 6 months), hereafter, be referred to as the “egg group” or “egg intervention group.” Baseline and endline (6 months following baseline) data were collected on socio‐economic and demographic factors, maternal and child anthropometry and child diet, morbidities, and blood biomarkers. No other contact was made with study participants following the end of Lulun Project until the start of Lulun II, approximately a 2‐year timeframe.

In Lulun II, the study team recontacted mothers or other caregivers to invite them to participate in the longitudinal follow‐up. They were first called on mobile phones with information from Lulun Project and then visited at households if phone contact was unsuccessful. Documentation of reasons for losses to follow‐up were obtained from participants, family members, or neighbours when original participants could not be located. Community meetings were held with community leaders and representatives to explain the follow‐up study and field team activities. The study was reviewed and approved by the ethics committees of Washington University in St. Louis and Universidad San Francisco de Quito. Written informed consent was obtained from mothers or other caregivers during household visits prior to commencement of data collection. Data collection was then performed at the house of the child or in preschools.

### 2.2 Procedures

A 3‐day training was conducted with the study team that included Lulun Project's investigators, the field team, and students from Washington University in St. Louis. The training consisted of theoretical foundations in nutrition, bone health, and social marketing, followed by practice in survey administration, anthropometry, and ultrasound techniques for bone age. Methods were pilot tested and validated against gold standard techniques during the training. Results from the bone health component of this trial will be reported elsewhere.

The study team collected all data in participant households from June to August, 2017. Questionnaires were administered to collect information on socio‐economic and demographic factors; 24‐hr frequency of dietary intakes by children; 2‐week morbidity recalls for fever, diarrhoea, respiratory conditions, and other health problems; and factors associated with the social marketing strategy conducted during the Lulun Project. A minimum dietary diversity score was generated based the child consuming four or more of the following food groups: (a) grains, roots, and tubers; (b) legumes and nuts; (c) dairy products (milk, yogurt, cheese); (d) flesh foods (meat, fish, poultry, and liver/organ meats); (e) eggs; (f) Vitamin A–rich fruits and vegetables; (g) other fruits and vegetables (World Health Organization [WHO], 2010).

Anthropometric measures for height, weight, and head circumference were made using WHO Growth Standard protocols (WHO, 2006). Pairs of enumerators took two measures of height to the nearest 1 mm, and a third if measures were greater than 5 mm apart, using a stadiometer (Seca GmbH & Co KG, Hamburg, Germany). Two measures of weight were taken using Seca Model 874 Electronic Digital Scale (Seca GmbH & Co KG, Hamburg, Germany), and third when measures differed by 0.05 kg. Finally, head circumference was measured twice using ShorrTape© Measuring Tape (Weight and Measure, LLC, Maryland USA), and third taken when differences in measures exceeded 2 mm. In the original Lulun Project trial, child length was measured in supine position and standardized to LAZ as per WHO protocol for children less than 2 years of age. Anthropometric measures were transformed to *z* scores: HAZ, weight‐for‐age (WAZ), weight‐for‐height (WHZ), body mass index (BMIz), and head circumference‐for‐age (HCZ); stunting was defined as HAZ < −2 and underweight WAZ < −2 (WHO, 2006).

### 2.3 Statistical analyses

We followed a similar analytical plan as in the Lulun Project. Univariate analyses were first performed to test for differences in the participant characteristics by group to ensure comparability using *t* test and chi square. Although not recommended in the reporting of an RCT (Schulz, Altman, & Moher, 2010), this was a follow‐up cohort study with potential bias in losses to follow‐up by group, so it was important to test differences in characteristics for comparability across original study groups.

Anthropometric outcomes from Lulun II were compared first by group, adjusting for the same covariates as were used in the Lulun Project (age, sex, and corresponding baseline anthropometry). Corresponding baseline anthropometry was included in models because there were differences by group at baseline Lulun Project. WHZ score was not included because wasting was not prevalent in this population (1 case at Lulun Project baseline, and 0 cases at Lulun Project endline). We also generated variables for change in anthropometry between the Lulun Project endpoint to Lulun II in order to examine differences in growth trajectories by group. Change in anthropometry from Lulun Project baseline to Lulun II was also assessed to investigate the egg effect over the entire intervention and postintervention period. Consistent with the Lulun Project analytical plan, we applied generalized linear modelling and log binomial models. However, these models showed no difference from simple linear and logistic regression modelling, which are reported here.

A second set of exploratory models were generated to investigate factors predicting the anthropometric outcomes in this older cohort. We tested all variables with a theoretical basis for driving growth. Factors shown to be associated (*p* < .05) with anthropometric outcomes in regression models adjusting for group, baseline anthropometry, and age, were then tested for mediation using structural equation modelling.

The original sample size calculations were based on hypothesized effect size for biomarker outcomes (difference in difference with control) of 0.35 assuming a 20% attrition rate (Jones et al., 2007 & Taneja et al., 2013). We recruited 163 mother or other caregiver–infant dyads and had a 9% loss to follow‐up (*n* = 148 analysed at the endline of Lulun Project), which produced a sufficient size to detect a 0.63 difference in HAZ. For Lulun II, there was an 11% loss to follow‐up, or 20% total from baseline. Applying the *SD* = 0.9 of HAZ from Lulun II, we were powered to detect 0.45 HAZ difference (*α* = .05; *β*‐1 = .80).

Data analyses were performed with Stata software (version 13.1; StataCorp, College Station, TX). The trial is registered at http://clinicaltrials.gov, identifier NCT02446873.

## 3 RESULTS

**Figure 1:**
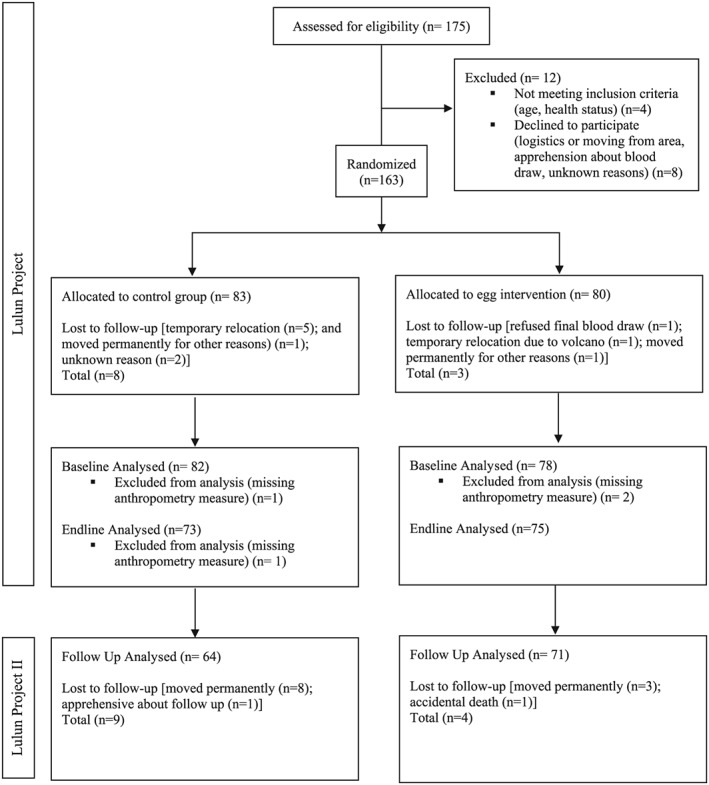
Flow diagram of Lulun Project and Lulun Project II in Cotopaxi Province, Ecuador. For the original Lulun Project randomized controlled trial, mother (caregiver)‐child pairs were recruited and enrolled in March–June 2015. They were followed for 6 months during the intervention period and data collected again at endline, September–December 2015. The same mother (caregiver)–child pairs were recontacted, recruited, and enrolled in May 2017 for Lulun Project II. Data on socio‐economic and demographic status, child diet, morbidities, and anthropometry were collected for the follow‐up cohort study, Lulun Project II, from May–August 2017.

Of the 148 children completing data collection at the endline of the Lulun Project, the study team reached 140 mother or other caregiver–infant dyads and recruited and enrolled 135 in the Lulun II (Figure 1, flow diagram) constituting a 9% loss to follow‐up from the endline of the Lulun Project, and 17% from baseline Lulun Project. We observed evidence for differential losses to follow‐up, 12% losses in control group versus 5% losses in egg group from endline of the Lulun Project. The primary reason for the loss was that some families had moved from the area. Only one parent from the control group declined participation citing concerns about the blood draw from the Lulun Project.

No differences were observed across characteristics between egg and control group children in Lulun II (Table 1). Across all children, 28.8% of respondents were not the mother of the index children, although 8.2% were fathers and 98.6% of respondents indicated that they were the primary caregiver. Stunting was highly prevalent across all children (50%) and showed no difference by group. Underweight affected only seven children, and there were no cases of low BMIz. The percentage of mothers or caregivers reporting ownership of poultry was significantly higher in the egg intervention group (76%) compared with the control group (56%; *p* = .02), a difference not observed in the Lulun Project. Among those owning poultry, we observed high variability in the numbers of chickens owned, although the mean number of chickens owned did not differ by group, egg intervention group (7.35 ± 7.02) versus control (6.39 ± 8.4; *p* = .50). There were 20% of children reported to have had acute diarrhoea in the previous 2 weeks and over half (52%) with congestion or runny nose morbidities but no differences evident by group.

**Table 1:** Characteristics of Lulun Project II sample, by original study trial arm

Characteristic	Control (*n* = 64)	Egg (*n* = 71)
Child		
Age, months (*SD*)	34.0 (1.8)	33.8 (1.8)
Female (%)	32 (50.8)	29 (41.4)
Mother (caregiver)		
Mother of index child as respondent (%)	48 (75.0)	48 (67.6)
Mother employed outside home (%)	32 (50.0)	34 (47.9)
Household		
Total household members	5.4 (1.9)	6.1 (2.6)
Total children under 5 years in household	1.4 (0.5)	1.5 (0.8)
Food production (%)	50 (78.1)	49 (69.0)
Livestock ownership (%)	61 (95.3)	68 (95.8)
Child morbidities, 2‐week recall		
Acute diarrhoea (%)	14 (22.9)	13 (18.3)
Skin rash (%)	13 (20.3)	10 (14.1)
Cough (%)	29 (45.3)	34 (48.6)
Congestion or runny nose (%)	35 (54.7)	35 (49.3)
Difficulty breathing (%)	7 (10.9)	11 (15.5)
Acute respiratory infection (%)	6 (9.4)	10 (14.1)

*Note*. Values are means ± standard deviations (*SD*) and percentages (%).

There were more mothers or caregivers who reported their child consumed any eggs in the previous 24 hr in the egg group (70.4%) compared with the control group (57.8%), although this difference did not reach significance (*p* = .13, by Pearson's chi square). No difference in the number of eggs consumed by control or egg group was observed in the previous 24 hr (1.55 ± 0.99) or in the previous 7 days (5.28 ± 3.09). We did observe a significant difference in the method of egg preparation, specifically, the number of fried eggs consumed in the previous week: egg (1.73 ± 2.14) and control (1.02 ± 1.12; *p* = .018, by Student's *t* test). There were no differences between groups for the other egg preparation methods—hard boiled or omelette. The percentage of children consuming ASF in the previous 24 hr showed no differences by group: milk (38.5%), fish (17.0%), and meat (77.8%). No other food types were significantly different by group except salty snack consumption in the control group (35.9%), which was higher than egg group (19.2%; *p* = .04). The percentage of children meeting minimum dietary diversity (83.7%) and mean seven‐item dietary diversity score (4.78 ± 1.16) also did not differ by group.

**Figure 2:**
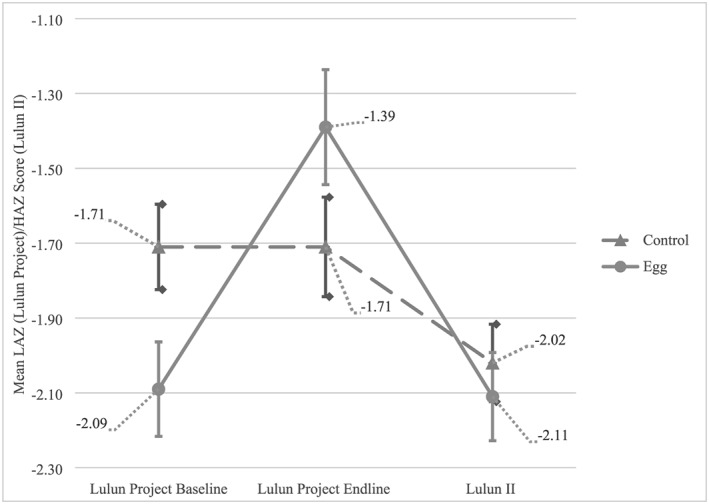
Mean length‐for‐age *z* scores (Lulun Project Baseline, Lulun Project Endline) and height‐for‐age *z* scores (Lulun Project II). The figure graphically represents the change in mean *z* scores for the egg intervention group (solid line) and control group (dashed line) from baseline Lulun Project (March–June 2015) to endline Lulun Project (September–December 2015) to Lulun Project II (May–August 2017). Standard error bars are presented for the measures at different time points, by group.

Growth faltering occurred in both groups from the endline of the Lulun Project (Figure 2). In Lulun II one half of all the children in the sample were stunted; mean HAZ was −2.07 ± 0.91. Only 5.3% of children were underweight, and the percentage of children with high BMIz > 2 was 5.3%, with a mean BMIz of 0.82 ± 0.79. Regression modelling showed no group effect across all models for HAZ, WAZ, BMIz, and HCz (Table 2). Children in the egg group showed a significant reduction in HAZ from Lulun Project endline to Lulun Project II compared with the control group after adjusting for age and baseline HAZ.

**Table 2:** Regression models of Lulun Project II anthropometric outcomes

Anthropometric outcome	Mean *z* scores for anthropometric outcomes with standard deviation (*SD*), by group				*β* coefficient for group effect	Group effect regression models		
	Control (*n* = 64)	*SD*	Egg (*n* = 71)	*SD*		95% CI	*p* value	Overall model Adjusted *R*
Height‐for‐age *Z* 1	−2.02	0.81	−2.11	0.98	.082	[−0.148, 0.313]	.482	.468
Weight‐for‐age *z* 1	−0.68	0.71	−0.86	0.77	.042	[−0.153, 0.238]	.671	.447
Body mass index *z* 1	0.89	0.75	0.76	0.82	−.018	[−0.282, 0.246]	.892	.112
Head circumference *z* 2	−0.37	0.79	−0.40	0.83	−.018	−0.294, 0.258	.896	.016
Difference in height‐for‐age *z* at Lulun Project II from length‐for‐age *z* at Lulun Project endline^1^	−0.29	0.84	−0.73	0.90	−.497	[−0.781, 0.212]	.001	.172

1 Linear regression models for group effect on anthropometric outcomes, adjusted for child baseline age and baseline anthropometry.

2 Linear regression models for group effect on anthropometric outcomes, adjusted for child baseline age.

In the second set of exploratory models, we identified factors associated with the anthropometric outcomes that improved models, based on adjusted *R* ^2^ (Table 3). Any egg consumption in the previous 24 hr was associated with reduced declines in HAZ from endline Lulun Project to Lulun Project II. Fever with acute diarrhoea negatively correlated with WAZ, whereas fever morbidity negatively correlated with BMIz. Household size showed an inverse relationship to HCz, adjusting for group assignment and child age. These variables were then examined for mediation in the event that group effect acted indirectly through mediating diet or morbidity factors. Only egg consumption showed a trend for significant mediation for change in HAZ, explaining 8.8% of the total effect.

**Table 3:** Determinants of anthropometric outcomes in Lulun II^1^

	Change in HAZ from Lulun I endline			HAZ Lulun II			WAZ Lulun II			BMIz Lulun II			HCZ Lulun II		
Determinants	Regression coefficient	Std. error	*p* value	Regression coefficient	Std. error	*p* value	Regression coefficient	Std. error	*p* value	Regression coefficient	Std. error	*p* value	Regression coefficient	Std. error	*p* value
Child age, months	.105	0.040	.010	.059	0.033	.076	−.002	0.027	.790	−.043	0.035	.226	.017	.036	.639
Household size^2^	–	–	–	–	–	–	–	–	–	–	–	–	−.071	.029	.017
HAZ baseline	−.291	0.072	<.001	.606	0.060	<.001	–	–	–	–	–	–	.317	.074	<.001
WAZ baseline	–	–	–	–	–	–	.447	0.044	<.001	–	–	–	–	–	–
BMIz baseline	–	–	–	–	–	–	–	–	–	.295	0.060	<.001	–	–	–
Any eggs, 24‐hr recall^3^	.330	0.151	.031	–	–	–	–	–	–	–	–	–	–	–	–
Any milk, 24‐hr recall^3^	–	–	–	–	–	–	–	–	–	−.275	0.128	.033	–	–	–
Fever, 2‐week recall^4^	–	–	–	–	–	–	–	–	–	−.563	0.156	<.001	–	–	–
Fever with diarrhoea, 2‐week recall^4^	–	–	–	–	–	–	−.829	0.250	.001	–	–	–	–	–	–
Adjusted *R* ^2^	.20	–	<.001	.45	–	<.001	.47	–	<.001	.18	–	<.001	.18	–	<.001

Abbreviations: BMIz, body mass index *z* score; HAZ, height‐for‐age *z*; HCZ, head circumference *z* score; WAZ, weight‐for‐age *z* score.

1 All linear regression models were adjusted for group effect.

2 Household size defined as total number of people living in the same household.

3 Any dietary intake of the food item by the child in the previous 24 hr.

4 Morbidity experienced by the child in the previous 2 weeks.

## 4 DISCUSSION

In this longitudinal follow‐up cohort study to the Lulun Project, we found that the growth effects of the egg intervention during the early complementary feeding period (6–15 months) were no longer evident 2 years later. We found that losses in HAZ were significantly greater among children in the egg group from endline Lulun Project to Lulun Project II, compared with the control group in regression modelling. Over 80% of the original baseline sample and over 90% of the endline sample from the Lulun Project participated in Lulun II. Any dietary intake of eggs was associated with reduced growth faltering from endline Lulun Project to Lulun II among all the children. Fever and fever with diarrhoea negatively associated with BMIz and WAZ, respectively. In our view, these findings speak to the need for high quality nutrition throughout early childhood to buffer the environmental effects in low‐resource contexts.

We showed the egg intervention effect on linear growth was no longer present after 2 years, but our findings suggest eggs continued to be associated with smaller postintervention decline. More children in the original egg group (70%) were reported to be consuming eggs in the previous 24 hr than in the control group (58%), pointing to a partial mediating effect in the change from LAZ measured at endline Lulun Project to HAZ measured for Lulun II from this study. This might be explained by changed feeding behaviours and food preferences for eggs instilled early in the complementary feeding period, by increased value for eggs in households through their observed positive effects, or potentially by messages conveyed in the social marketing campaign.^5^ In statistical terminology, the postintervention egg consumption might also be described as suppressing the postintervention HAZ decline (MacKinnon et al., 2000). However, the findings clearly show that losses in HAZ were significantly greater from endline Lulun Project to Lulun Project II in the egg group compared with control. Once consistent daily egg nutrition was removed from the diets, the losses were dramatic and other factors again influenced growth trajectories.

Few other longitudinal cohort studies have investigated the lasting effects of early food interventions in low‐resource countries on growth. One recent study in Bangladesh of the Rang‐Din Nutrition project examined the lasting effects from a four‐arm trial of different lipid‐based nutrient supplements during pregnancy, lactation, and early childhood 6–24 months (Dewey et al., 2017). Only subgroup analyses by sex of the child or level of household food insecurity showed continued differences among children followed 16‐ to 28‐month postintervention. In a widely cited trial in Guatemala during the 1970s (Ramirez‐Zea, Melgar, & Rivera, 2010), children received nutritional supplements of either atole (grain‐based, containing milk powder and protein gruel) or fresco (sugar drink) from birth to 7 years. Follow‐up studies suggest ongoing effects on school‐aged cognition and adult productivity and most recently, reduced odds of diabetes, among other health outcomes (Engle & Fernández, 2010; Ford et al., 2018; Hoddinott, Maluccio, Behrman, Flores, & Martorell, 2008; Martorell, 2010). Conditional cash transfer programmes with food supplementation represent another approach that has been examined longitudinally with some evidence for positive impacts with longer interventon periods.^9^ Finally, follow‐up studies to single or multiple nutrient supplementation trials suggest some long‐term impacts on metabolism and child health outcomes (Christian et al., 2009; Christian et al., 2010; Stewart et al., 2009; Stewart, Christian, LeClerq, West, & Khatry, 2009). In summary, our review of the literature indicates a limited evidence base for long‐term impacts arising from interventions during the first 1,000 days of life from experimental trials (Adair et al., 2013; Victora et al., 2008).

### 4.1 Growth faltering and plasticity: Beyond 1,000 days

Although malnutrition problems occur through the life cycle (ACC/SCN, 2000), the recent focus on programming and policy has been on the first 1,000 days of life. Indeed, we targeted children 6–9 months in the Lulun Project because global trends for stunting show the highest risks during early complementary feeding period (Victora, de Onis, Hallal, Blossner, & Shrimpton, 2010). However, data from Lulun Project II strongly suggest the risk for linear growth faltering remains at 3 years and may necessitate longer intervention periods. Other studies support this conclusion. Evidence from the longitudinal studies of the Consortium of Health Oriented Research in Transitioning Countries (COHORTS) Collaboration demonstrates the plasticity of linear growth beyond 2 years (Prentice et al., 2013). With only one country exception, India, data from Brazil, Guatemala, Philippines, and South Africa revealed fluctuations in HAZ through adolescence, indicative of rebounding growth. In rural Gambia, HAZ was assessed cross‐sectionally for over 60 years, showing common growth patterns across time: HAZ dropped until 24 months of age; catch‐up growth occurred until 5 years; there was loss again in early adolescence at the onset of puberty; and a slow regain observed until adult stature was achieved (Prentice et al., 2013). Again, few interventional studies, particularly of food‐based interventions, have contributed to our understanding of nutrition's influence on linear growth beyond 2 years of age (Roberts & Stein, 2017).

### 4.2 Context: Poverty and environmental factors

Beyond the main effect models, we examined other factors associated with anthropometric outcomes in Lulun Project II. Fever and fever with diarrhoea morbidities negatively correlated with WAZ and BMIz, respectively. The egg intervention early in the complementary feeding period may have mitigated the influence of negative environmental exposures, but after the 6‐month intervention period, children were no longer provided eggs regularly from the project to buffer these effects. We found that one in five children (20%) had acute diarrhoea in the previous 2 weeks as reported by the mother or caregiver, which is a relatively high prevalence compared with other populations regionally and globally.

Recent evidence from WASH intervention trials with and without nutritional supplementation have shown limited effects on reductions in diarrhoea and increased linear growth (Luby et al., 2018; Null et al., 2018). This may be due to community‐level exposures requiring long‐term mitigation. Poverty and limited access to high‐quality diets have been shown to correlate with stunting (Black et al., 2013; Danaei, Andrews, & Sudfeld, 2016; Victora et al., 2008). ASF in particular may be expensive and hard to access for low‐resource households (Headey & Alderman, 2019; Makrides, Hawkes, Neumann, & Gibson, 2002).

### 4.3 Transient effects

Multiple pathways drive growth in the human body. One study found over 200,000 single nucleotide polymorphisms explained only 45% of variance in the heritability in height (Yang et al., 2010). There is variability by context in terms of the proportion of height explained by genetic proxies, such as mid‐parental height (Garza, Borghi, Onyango, & de Onis, 2013). Eggs provided the nutrients and bioactive factors necessary to propel linear growth during the 6‐month intervention and potentially programme expectations in growth pathways. Once the egg intervention was removed, children likely returned to usual diets and potentially growth‐supressing environments resulting in great declines in HAZ.

In the Lulun Project, choline, betaine, and methionine were significantly increased in the egg compared with control, suggesting effects on the one‐carbon pathway and in part mediated the linear growth effect (Iannotti, Lutter, Waters, et al., 2017). Depending on storage pools, these nutrients may be required more consistently in the diet compared with others. Further, given the pathway that seemed to be affected, methylation or longer term programming may have altered the biological requirements for egg nutrients supplied on a regular basis. Once removed from the child's daily diet, the response we observed showed significant losses in HAZ. Targeted metabolomic analyses of blood biomarkers was not carried out in Lulun II due to insufficient resources, although it may have been interesting to explore lingering effects from these biomarkers including altered methylation patterns.

This study had some limitations. Although losses to follow‐up were minimal and not shown to influence comparability of the groups, we may have had insufficient numbers to detect impacts on all outcomes. There were differential losses to follow‐up with greater losses found for the control group (12%) compared with the egg group (5%) from endline Lulun Project. Again, our analyses showed this did not disrupt comparability in groups. Finally, there may have been a risk of regression to the mean, in view of the baseline Lulun Project differences in HAZ by group and the longitudinal design (Barnett, van der Pols, & Dobson, 2005). In our view, however, the RCT design minimizes the risk, and our findings for improved biomarkers, which did not differ at baseline Lulun Project, served to validate growth outcomes.

## 5 CONCLUSIONS

The Lulun Project II cohort study successfully followed over 90% of the children completing the Lulun Project's original trial. The effect from the egg intervention in the Lulun Project was no longer present in children now 2–3 years, and importantly, there were significant declines in HAZ for the egg group compared with the control group. Current egg consumption seems to have mitigated some of the losses in HAZ, but other factors such as diarrhoeal morbidities likely impaired growth in the postintervention period from 12–36 months of age. In our view, this finding speaks to the need for longer intervention periods and holistic stunting prevention approaches. Eggs can provide a critical bridge to bolster growth in highly vulnerable periods, but ongoing attention to overall diet quality and supportive environmental conditions should be sustained as a child ages.

## CONFLICTS OF INTEREST

The authors declare that they have no conflicts of interest.

## CONTRIBUTIONS

LI, WW, JN. CAGR, CL, and CS, designed the research. WW, JN, CAGR, PM, KD, and DH, conducted the research. LI, CS, CL, YC, and MC, analysed the data. LI and MC wrote the paper. LI had the primary responsibility for final content. All authors read and approved the final manuscript.
